# Positive Airway Pressure Therapy Adherence with Mask Resupply: A Propensity-Matched Analysis

**DOI:** 10.3390/jcm10040720

**Published:** 2021-02-12

**Authors:** Adam V. Benjafield, Liesl M. Oldstone, Leslee A. Willes, Colleen Kelly, Carlos M. Nunez, Atul Malhotra, on behalf of the medXcloud Group

**Affiliations:** 1 ResMed Science Center, San Diego, CA 92123, USA; adam.benjafield@resmed.com.au (A.V.B.); lmoldstone@gmail.com (L.M.O.); carlos.nunez@resmed.com (C.M.N.); 2 Willes Consulting, Encinitas, CA 92024, USA; lesleew@willesconsulting.com; 3 Kelly Statistical Consulting, Carlsbad, CA 92011, USA; kstat.consulting@gmail.com; 4 Pulmonary, Critical Care and Sleep Medicine, University of California, 9300 Campus Point Drive, La Jolla, San Diego, CA 92037, USA

**Keywords:** positive airway pressure, adherence, leak, patient engagement, sleep apnea, lung

## Abstract

There are currently few data on the impact of mask resupply on longer-term adherence to positive airway pressure (PAP) therapy. This retrospective analysis investigated the effects of mask/mask cushion resupply on the adherence to PAP versus no resupply. Deidentified patient billing data for PAP supply items were merged with telemonitoring data from Cloud-connected AirSense 10/AirCurve 10 devices via AirView^TM^ (ResMed). Eligible patients started PAP between 1 July 2014 and 17 June 2016, had ≥360 days of PAP device data, and achieved initial U.S. Medicare adherence criteria. Patients who received a resupply of mask systems/cushions (resupply group) were propensity-score-matched with those not receiving any mask/cushion resupply (control group). A total of 100,370 patients were included. From days 91 to 360, the mean device usage was 5.6 and 4.5 h/night in the resupply and control groups, respectively (*p* < 0.0001). The proportion of patients with a mean device usage ≥4 h/night was significantly higher in the resupply group versus the control group (77% vs. 59%; *p* < 0.0001). The therapy termination rate was significantly lower in the resupply group versus the control group (14.7% vs. 31.9%; *p* < 0.0001); there was a trend toward lower therapy termination rates as the number of resupplies increased. The replacement of mask interface components was associated with better longer-term adherence to PAP therapy versus no resupply.

## 1. Introduction

Obstructive sleep apnea (OSA) is a common disorder with major neurocognitive and cardiometabolic sequelae [1]. Recent estimates suggest that the number of people worldwide with OSA is up to 1 billion [2]. This finding highlights the importance of raising awareness of OSA and emphasizes the need for efficient approaches to large-scale diagnosis and treatment. The use of oral appliances or upper airway surgery are potential options for the treatment of OSA, but are limited by their variable efficacy and a relative lack of outcome data [3,4,5,6]. The current treatment of choice for OSA is positive airway pressure (PAP) therapy, which has been shown to improve symptoms, blood pressure, and quality of life in randomized controlled trials [7,8,9,10]. However, treatment is often suboptimal due to variable adherence to PAP therapy [11,12]. Adherence with PAP therapy is an important criterion for continuing treatment and is necessary for the benefits of therapy to be realized [11]. Therefore, considerable emphasis has been placed on optimizing adherence to PAP therapy [13,14].

Telemedicine strategies offer the possibility of remotely monitoring PAP therapy adherence and delivering interventions that are designed to improve device usage. We have recently reported that the utilization of new technology might contribute to improved device usage and a higher proportion of patients meeting the U.S. Center for Medicare and Medicaid Services (CMS, Woodlawn, MD, USA) PAP adherence criteria [13]. In our analysis using a propensity matching design, adherence in PAP users provided with a patient engagement tool was significantly higher than that in those managed with usual care monitoring (87% vs. 70%; *p* < 0.0001) [13].

The use of new technology, such as the patient engagement tool in the study described above, is a novel and compelling approach for improving adherence. In addition, basic contributors to good quality care, such as appropriate patient follow-up and supply replenishment, may also play a role in ensuring adherence with PAP therapy. Of note, some providers have advocated for regular changes of masks, hoses, and filters to optimize adherence [15]. Although financial incentives have driven some companies that produce durable medical equipment to provide regular replacement supplies, others might suggest that frequent replacement supplies may not be necessary if the masks are regularly cleaned and maintained. Payers may also limit access to supply items if they do not receive objective confirmation of usage requirements being met as frequently as every 3 months [16]. Patel et al. have reported mask refill rates as a predictor of PAP adherence, but the study used a modest sample size [17]. In clinical practice, many patients forget or lose track of the age of their equipment and supplies, making the optimal timing and approach to resupply unclear.

This study investigated the effects of the resupply of PAP equipment (mask system and/or cushions) on the adherence to PAP therapy compared with no resupply in the first year of therapy. The aim was to test the hypothesis that mask resupply would be associated with improved adherence to PAP therapy versus no resupply.

## 2. Methods

### 2.1. Study Design and Participants

This retrospective analysis merged deidentified patient billing data for PAP supply items (Brightree, Peachtree Corners, GA, USA) with telemonitoring data collected from Cloud-connected AirSense 10 and AirCurve 10 (ResMed, San Diego, CA, USA) devices via AirView^TM^ (ResMed, San Diego, CA, USA), a password-protected Cloud-based technology that is compliant with the Health Insurance Portability and Accountability Act. These anonymized data were sent to third-party independent statisticians who assisted with the analyses and presentation of findings. All patients had registered to use AirView^TM^ and provided consent for their data to be used in future analyses. The study protocol was reviewed by the Chesapeake institutional review board (IRB) and was deemed exempt from IRB oversight per the Department of Health and Human Services regulations 45 CFR 46.101(b) (4).

Eligible patients were identified in Brightree as having had initiated PAP therapy between 1 July 2014 and 17 June 2016, had the potential for at least 360 days of AirView^TM^ data available, and had achieved initial CMS adherence (defined as PAP device usage of ≥4 h/night on ≥70% of nights in a consecutive 30-day period in the first 90 days of therapy) were analyzed. Patients were excluded if they met the following criteria: multiple therapy start dates, use of multiple devices by the same subject, invalid or missing device serial numbers recorded, and the replacement of a patients’ continuous positive airway pressure (CPAP) device within 1 year of therapy initiation. Patient data were then merged with the AirView^TM^ data to obtain CPAP daily usage data. Further exclusions were applied as follows: incorrect device serial numbers listed, the same device was used by multiple patients, did not have the potential for 365 days of usage data, did not achieve the CMS 90-day adherence criteria, and date or data entry inconsistencies. These inconsistencies included: the therapy start date was missing or after the first usage date, therapy start date >3 days before the first usage date, and myAir^TM^ registration date >3 days before or >90 days after therapy start date.

Patients were divided into two groups: one including those receiving mask resupply (mask system and/or mask cushion; resupply group) and the other including those who did not receive mask resupply in the first year of therapy (control group) as part of their standard care. To minimize the bias in between-group comparisons, a propensity model was constructed using the following baseline variables to match patients in the resupply and control groups: gender, age at initiation of the PAP therapy, therapy start date, mode of PAP therapy, 95th percentile mask leak on day 1, residual apnea–hypopnea index (AHI) on day 1, and use of a patient engagement strategy (myAir^TM^). Estimated propensity scores were then used to form 1:1 matched pairs, with the propensity scores being within a window of 5% being matched.

### 2.2. Endpoints

The primary objective was to compare the adherence with PAP therapy (defined in terms of the average hours of use per day) on days 91–360 in the resupply and control groups. Secondary objectives were the proportion of patients with a mean PAP usage of ≥4 h/day, the proportion of patients who stopped using PAP, average unintentional leaks, average residual AHI, and the frequency of resupply distributions in the resupply group. All PAP-related data were obtained from AirView^TM^.

### 2.3. Statistical Analysis

The minimum sample size was set at 3800 (1900 per group with 1:1 matching) to provide an 80% probability of detecting a 0.2 h difference in the mean usage between the resupply and control groups, assuming a standard deviation of 2.2 h in each group (as was observed in a pilot study). The required sample size was calculated using the two-sample *t*-tests assuming equal variance procedure with NCSS Power Analysis Statistical Software, version 14 (Kaysville, UT 84037, USA), based on a two-sided, two-sample *t*-test. This sample size based on independent groups was expected to be conservative because the two groups were matched for baseline characteristics, reducing the variance of the difference and yielding greater power.

The primary analysis population included all patients fulfilling the inclusion and exclusion criteria and matched in the propensity score procedure. Descriptive statistics were used to present the demographic and therapy characteristics for the primary analysis population, including age, sex, myAir^TM^ use, and device mode. Descriptive statistics were also used to present the mean usage, the proportion of patients with mean usage of ≥4 h/night, the proportion of patients with a usage of ≥4 h/night on 70% of nights, the mean AHI, the median 95th percentile mask leak between 91 and 360 days after initiation of therapy, and the proportion of patients who terminated therapy (i.e., PAP therapy termination, as studied previously) [18,19,20]. Continuous parameters were compared between groups using a mixed-effect linear model, with the pair identifier as a random effect and the resupply/control group as a fixed effect. Percentages were compared using the McNemar test. Kaplan–Meier analysis was used to compare survival functions for the resupply and control groups, where survival was defined as remaining adherent to PAP therapy; median adherence times in the two groups were compared using a log-rank test. Patients were considered to have terminated their therapy if their total usage was 0 h during a 30-day window. Kaplan–Meier analyses were also used to investigate the differences in survival probabilities, which was stratified by the number of resupplies received within the first year.

Sensitivity analyses included matching the resupply and control patients using a 0.2 window of the linear propensity scores, as suggested by Rubin [21], and checking the three conditions mentioned therein for regression adjustment methods to be reliable: (1) the means of the propensity scores in the two groups are similar, (2) the standard deviations of the propensity scores in the two groups are similar, and (3) the residual variances of the covariates after adjusting for the propensity score in the two groups are similar.

A piecewise linear model was fit to each of 1000 randomly selected patients’ usage data 30 days prior to and 30 days after their first mask resupply following the initial 90 days of usage. Usage data before and after resupply was modeled with separate linear models. A mixed-effect piecewise linear regression model was used to describe daily usage data between 30 days prior and 30 days after the first resupply. The explanatory variables were the random slope and intercepts before resupply (each patient had their own slope and intercept) and the random slope and intercepts after resupply. An AR (1) auto-correlation was assumed for successive daily usage data. The total difference in usage (over 30 days after first resupply) between the predicted usage after resupply and the usage predicted if the before-resupply trend continued was calculated for each subject and then averaged over subjects.

Any *p*-values < 0.05 were considered statistically significant. All statistical analyses were performed using SAS version 9.3 (Cary, NC 27513, USA) or later. Graphics were generated using SAS software.

## 3. Results

### 3.1. Study Sample

**Figure 1:**
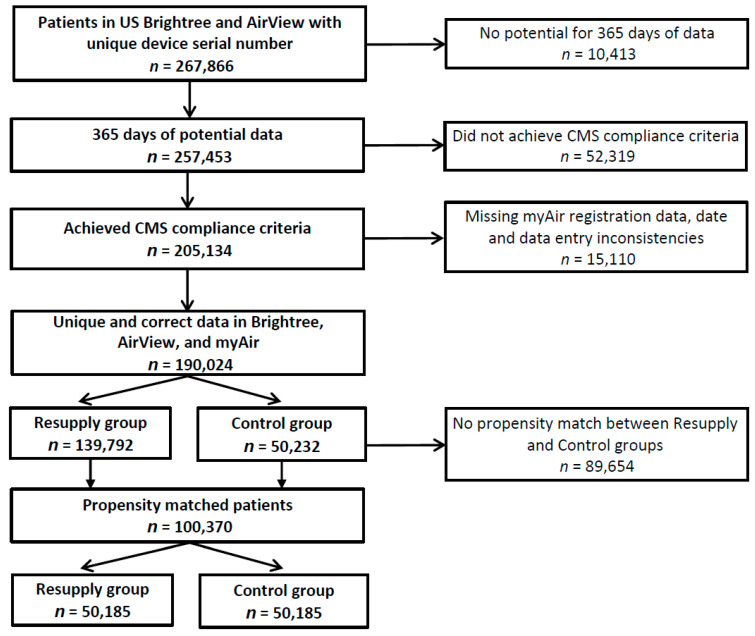
Flow diagram. CMS, Centers for Medicare and Medicaid.

The analysis included a total of 100,370 patients (mean age 57 years, 64% male) (Table 1). A flow diagram showing the patient identification and selection is shown in Figure 1.

### 3.2. Resupply

Patients in the resupply group (*n* = 50,185) received a mean of 5.0 ± 4.5 (median 3.0) items during the study period (mean 2.1 ± 1.5 (median 2.0) shipments per patient, with a mean of 2.4 ± 1.7 (median 2.0) items per shipment).

### 3.3. Adherence

**Figure 2:**
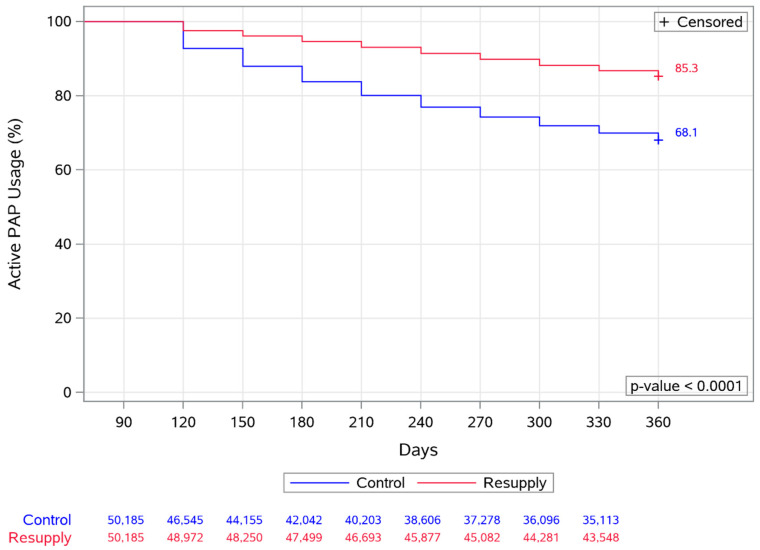
Overall probability of continuing with the positive airway pressure (PAP) therapy (“survival” of therapy) from days 91–360 in the control and resupply groups (primary analysis population). +Censored, patients whose therapy continued after 360 days were censored at the 360 day time point.

**Figure 3:**
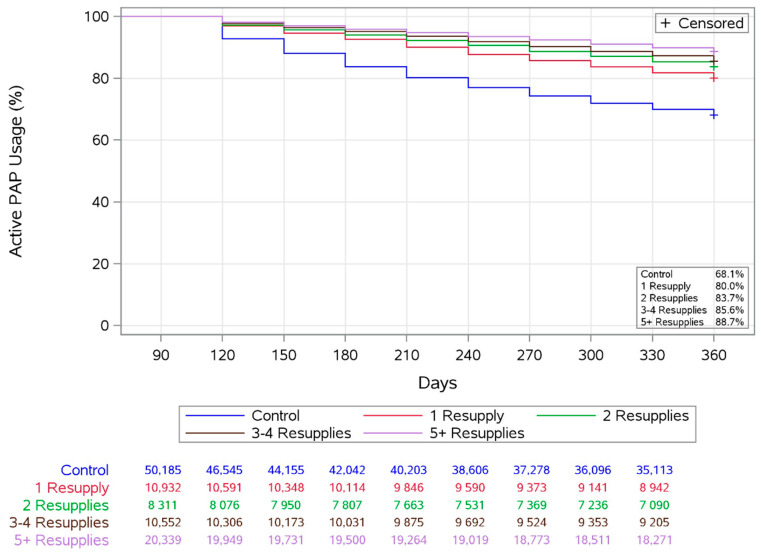
Probability of continuing with the positive airway pressure (PAP) therapy (“survival” of therapy) from days 91–360 in the control and resupply groups broken down by the number of resupplies shipped (primary analysis population). +Censored, patients whose therapy continued after 360 days were censored at the 360 day time point.

Adherence with the PAP therapy from day 91 to day 360 was significantly better in the resupply group versus the control group, including mean daily usage, the proportion of patients with mean usage ≥4 h, and the proportion of patients with daily usage ≥4 h for 70% of nights (all *p* < 0.0001) (Table 2). The overall probability of continuing with the PAP therapy during the study period (“survival” of therapy) was 85.3% in the resupply group and 68.1% in the control group (*p* < 0.0001) (Figure 2), corresponding to therapy termination rates of 14.7% and 31.9%, respectively. This finding was consistent across subgroups based on the number of resupplies shipped (Figure 3).

### 3.4. Respiratory Parameters

The mean AHI and median 95th percentile leak were slightly but statistically significantly lower in the resupply group compared with the control group (both *p* < 0.0001) (Table 2).

### 3.5. Sensitivity Analysis

The results were almost identical when subjects were matched using a linear score window of 0.2 instead of the propensity score window of 5%. Furthermore, Rubin’s three conditions for the reliability of regression adjustment methods [20] were all met: (1) the mean linear score (log-odds of the propensity score) was 0.98785 in the resupply group and 0.98787 in the control group, (2) the standard deviation of the linear score was 0.2667 in the resupply group and 0.2666 in the control group, (3) the ratios of the residual variances of the covariates adjusted for the propensity score in the resupply to the control group ranged from 0.991 to 1.003. Therefore, all three of Rubin’s conditions were met in the matched pairs analysis population.

### 3.6. Longitudinal Analysis

On average, patients’ usage decreased by 0.27 min per day before resupply and decreased by 0.13 min per day after resupply, which is an improvement that was statistically significant (*p* < 0.0001). The average total difference in usage over 30 days after resupply (the difference between the two estimated linear regression before and after resupply summed over the 30 days after resupply) showed an increase of 211 min with a 95% confidence interval of 112 to 311 min.

## 4. Discussion

This study showed that regular mask resupply was associated with better usage of the PAP therapy from 3 months to 1 year after treatment initiation. These findings are unique because, to the best of our knowledge, they represent the first major effort to evaluate adherence to PAP therapy as a function of mask resupply.

The average 1.1 h increase in PAP usage in the resupply group versus the control group is likely to represent a clinically relevant improvement [9,11,22]. Although the database used in this study did not allow for the determination of objective hard outcomes, published data suggest that this degree of improvement in device usage is associated with improvements regarding sleepiness, daily functioning, and blood pressure [9,11,22]. Another important finding is that rates of therapy termination, perhaps the worst form of non-adherence, were significantly lower in the resupply group compared with the control group. Maintaining patients on PAP therapy is an important clinical goal because retention increases the likelihood of achieving PAP-therapy-related benefits. The observation that therapy termination rates decreased as resupply volume increased was also of note. Our novel findings suggest that adequate levels of mask resupply might have a beneficial influence on long-term PAP therapy adherence.

To assess the possibility of reverse causation, we performed a longitudinal analysis using a random sample of 1000 patients before and after the first resupply occurring after the 90 initial days of usage. The possibility existed that ongoing PAP usage drove mask resupply rather than mask resupply yielding improved PAP adherence. A mixed effect piecewise linear regression model was used to define daily usage in the 30 days prior to and 30 days after the resupply event. By using a narrow time window, we alleviated some concern regarding the possibility that ongoing PAP usage drove an ongoing need for masks. Moreover, the improvements observed with resupply in this longitudinal analysis provide reassurance that we have identified important clinical findings that are likely to benefit patients rather than a statistical artifact of an observational study.

A number of interventions have been suggested to improve PAP adherence, although there are inconsistencies in the results between studies, leading to ongoing discussions [23]. Intensive support and education has been linked to good PAP adherence in some, but not all, studies [24]. Furthermore, patient engagement using new technology or other approaches may be beneficial [13]. Financial incentives and group PAP therapy have also been advocated for [25,26,27], although data in this area are limited. Variable success has been reported in clinical trials investigating the use of pressure relief, humidifiers, and various pressure modes on the adherence to PAP therapy [28,29,30]. In clinical practice, many patients express a preference for one device/mode over another [31], emphasizing the need for an individualized or personalized approach to PAP delivery. As part of this approach, our findings suggest that telemedicine-based patient support, including regular mask resupply, could contribute to improved adherence to PAP therapy.

There are small amounts of published data available to inform the optimal frequency of mask changes during the regular use of PAP. We are aware of efforts to assess the biofilm that can develop on masks and to perform quantitative cultures on the masks, which have suggested increased colony-forming units after 6 months of consistent use [32]. However, recent data suggest that the use of PAP delivered via a nasal mask or full face mask does not increase the risk of respiratory infection compared to controls with OSA who did not receive PAP [33]. Furthermore, even bacterial colonization of the CPAP humidifier reservoir was not associated with higher rates of chronic rhinosinusitis in regular PAP users [34].

In addition to theories about bacterial loads, another consideration might be the development of mask leaks, which tend to happen as masks get older. Clinical experience and some published data have shown that leaks can develop with ongoing PAP usage as masks age and that leaks are predictive of poor PAP adherence [35]. Thus, in theory, mask resupply may be beneficial for a number of reasons, although potential mechanisms are not yet clear based on the literature and the data available from our study.

Current CMS criteria allow for reimbursement of a new mask system every 3 months and two replacement mask cushions every month [16]. Based on these allowances, patients in our study could have accessed three new mask systems and 18 new cushions (i.e., 21 items in total) for the 9-month study period. Our findings documented an average number of 2.1 resupply shipments per patient with a mean of 2.4 items per shipment, meaning that the total resupply over the study period was about 5 items on average. This frequency is substantially below reimbursement limits and indicates that overall mask resupply is generally not being overutilized. In addition, it is possible that better use of mask resupply allowances could contribute to even better longer-term adherence to PAP therapy. Nevertheless, further work is needed to define the optimal frequency of mask changes during long-term PAP therapy. The optimal strategy may differ between patients based on individual preferences, local temperature and humidity, hours of usage, and other factors, such as the patient microbiome.

Several limitations need to be taken into account when interpreting the findings of this study. First, this was not a randomized clinical trial and therefore definitive conclusions about the causal effects of resupply cannot be drawn. However, propensity matching was used, where this approach helped to limit the contribution of confounding factors to the study results. Furthermore, our longitudinal analysis supported the hypothesis that resupply increased usage. The fact that our findings were independent of traditional predictors of PAP adherence suggests that resupply per se may be quite helpful. Second, we did not have any information about how patients managed their resupply or whether they were part of a formal resupply program. We only have data on supply items coded for reimbursement purposes and could not determine factors that led to the resupply behavior. Thus, we cannot exclude the possibility that patients in the resupply group differed from those in the control group in other important ways, including motivation or socioeconomic status, or some other unrecognized confounder [36]. While we acknowledge the potential impact of the “healthy user effect,” the same issue might also arise with randomized controlled trials in which PAP adherence may be a marker of a good prognosis. Finally, by design, our study included patients who had met CMS adherence criteria in the first 90 days of therapy, with the aim toward looking at longer-term adherence. As a result, those with poor early adherence who failed to reach CMS criteria were excluded but perhaps unlikely to benefit from a resupply program. Similarly, patients who did not have access to health care or refused PAP therapy would not have been included. Therefore, the effect of a resupply program on adherence in these patient groups is unknown, and our findings are only applicable to patients who have characteristics that are similar to the study population.

In conclusion, this big data analysis including a large group of well-matched patients treated with PAP therapy in routine clinical practice showed that reasonable resupply of mask interfaces was associated with statistically and clinically significant increases in average daily PAP device usage and lower rates of therapy termination compared with no resupply (control). These improvements in PAP therapy adherence and usage could positively impact clinical outcomes if confirmed in future randomized trials.

## Figures and Tables

**Table 1:** Demographics and therapy characteristics for the primary analysis population.

Heading	Control (*n* = 50,185)	Resupply (*n* = 50,185)
Age, years		
Mean ± *SD*	56.9 ± 13.6	57.0 ± 13.5
Median	57.0	57.0
Sex, *n* (%)		
Female	17,847 (35.6)	17,858 (35.6)
Male	32,312 (64.4)	32,301 (64.4)
Missing	26 (<0.1)	26 (<0.1)
Device/mode, *n* (%)		
APAP	21,437 (42.7)	21,449 (42.7)
CPAP	23,949 (47.7)	24,022 (47.9)
Bilevel	4272 (8.5)	4187 (8.3)
Missing	527 (1.1)	527 (1.1)
myAir^TM^ use, *n* (%)	8807 (17.5)	8795 (17.5)
AHI on the first day of therapy, /h		
Mean ± *SD*	3.9 ± 6.0	3.8 ± 5.7
Median	2.0	2.0
95th percentile leak on the first day of therapy, L/min		
Mean ± *SD*	23.5 ± 26.3	23.2 ± 25.7
Median	15.6	15.6

AHI, apnea–hypopnea index; APAP, automatically titrating continuous positive airway pressure; CPAP, continuous positive airway pressure; *SD*, standard deviation.

**Table 2:** Device usage and respiratory parameters from days 91 to 360 (primary analysis population).

Device Usage	Control	Resupply	*p*-Value
	(*n* = 50,185)	(*n* = 50,185)	
Mean usage, h			
Mean (*SE*)	4.5 (0.1)	5.6 (0.01)	
Median	4.9	6.0	
Mean difference vs. control (95% CI)		1.1 (1.06, 1.13)	<0.0001
Mean usage ≥4 h, %			
Mean (95% CI)	59.2 (58.8, 59.6)	77.0 (76.6, 77.4)	
Mean difference vs. control (95% CI)		17.8 (17.2, 18.3)	<0.0001
Daily usage ≥4 h for 70% of nights, %			
Mean (95% CI)	50.9 (50.5, 51.3)	67.7 (67.3, 68.1)	
Mean difference vs. control (95% CI)		16.8 (16.2, 17.4)	<0.0001
**Respiratory Parameters**	**(*n* = 47,373)**	**(*n* = 49,359)**	
Mean AHI, /h			
Mean (*SE*)	2.6 (0.02)	2.5 (0.01)	
Median	1.5	1.5	
Mean difference vs. control (95% CI)		−0.2 (−0.21, −0.13)	<0.0001
Median 95% percentile mask leak, L/min			
Mean (*SE*)	20.4 (0.10)	19.0 (0.09)	
Median	15.0	14.4	
Mean difference vs. control (95% CI)		−1.5 (−1.68, −1.24)	<0.0001

AHI, apnea–hypopnea index; CI, confidence interval; *SE*, standard error.

## Data Availability

The data are not publicly available due to privacy and patient consent limitations.
